# Stepwise Surgical Management of Persistent Pleural and Parenchymal Sepsis Due to Pan-Resistant Pseudomonas Infection

**DOI:** 10.3390/jcm15124711

**Published:** 2026-06-17

**Authors:** Konstantinos Kostopanagiotou, Valentina Karantana, Małgorzata Edyta Wojtyś, Elias Santaintidis, Nikolaos Korodimos, Nektarios I. Koufopoulos, Theofanis Nastos, Arkadiusz Waloryszak, Konstantinos Thomas, Periklis Tomos

**Affiliations:** 1 Thoracic Surgery Department, National and Kapodistrian University of Athens, School of Medicine, Attikon University Hospital, 12462 Athens, Greece; 2 4th Department of Internal Medicine, National and Kapodistrian University of Athens, School of Medicine, Attikon University Hospital, 12462 Athens, Greece; 3 Department of Thoracic Surgery and Transplantation, Pomeranian Medical University in Szczecin, Alfreda Sokołowskiego 11, 70-891 Szczecin, Poland; 4 2nd Department of Pathology, National and Kapodistrian University of Athens, School of Medicine, Attikon University Hospital, 12462 Athens, Greece; 5 Student Scientific Club of Thoracic Surgery and Transplantation, Pomeranian Medical University in Szczecin, 70-204 Szczecin, Poland

**Keywords:** pan-resistant Pseudomonas, pleural sepsis, necrotizing lung infection, necrotizing pneumonia, lung gangrene, thoracoplasty, thoracoscopic debridement, salvage lobectomy

## Abstract

Refractory thoracic infections require targeted antimicrobial combinations, repeated drainage interventions and often staged surgical procedures of varying complexity grades. In necrotizing pneumonia cases, successful treatment is complete removal of destroyed non-functional parenchyma, pleural cavity debridement, and complete pathogen eradication based on culture-based sensitivity-driven antimicrobials. The latter proves challenging in pan-resistant microbial strains where both medical and surgical treatments demonstrate limited effectiveness. We describe a case of persistent thoracic sepsis due to pan-resistant Pseudomonas receiving sequentially thoracoscopic decortication, thoracotomy for lobectomy, and open thoracostomy as the last treatment option to prevent fatal sepsis in view of non-available antibiotics. The immediate source-control effect raised the question of the ideal timing for selecting an aggressive thoracoplastic procedure despite its deforming nature over any other treatment approach.

## 1. Introduction

Neglected and mismanaged pneumonia patients may develop pleural empyema and necrotizing lung infections (NLIs) requiring treatment with empirical and culture-driven antimicrobials and a variety of interventions ranging from simple percutaneous drainage to complex high-risk thoracic procedures in severely complicated cases [1,2]. Over this course, an NLI may become a life-threatening situation, predisposing the patient to sepsis and even acute respiratory failure [3,4]. A more severe form is pulmonary gangrene with pulmonary vascular thrombosis, tissue liquefaction, uncontrolled bacterial replication, reduced antibiotic tissue penetration, and potentially the development of bronchopleural fistula or life-threatening hemoptysis [5,6,7,8]. Gangrene is diagnosed when vascular thrombosis and central parenchymal necrosis of more than 50% are identified on computed tomography CT scans [9,10]. A recent diagnosis of NLI, especially with abscesses or gangrene, should prompt early consultation with a thoracic surgeon for surgical exploration consideration [5]. The exact mortality rate of NLIs is unknown, but literature-reported rates close to 50% mean that it is a very high risk [7,9,11]. For adult NLIs, the commonest recognized pathogens are *Streptococcus pneumoniae*, *Staphylococcus aureus*, and *Klebsiella pneumoniae* [8,12]. In addition, Gram-negative pathogens, especially *Pseudomonas aeruginosa* and *Klebsiella pneumoniae*, have been associated with pulmonary gangrene and a poor prognosis [12,13,14]. Hospitalized patients generally receive empirical anti-pseudomonal protection while awaiting culture results for identification and antibiotic susceptibility testing [5,9,15,16]. Antibiotics with activity against *P. aeruginosa* include penicillin in combination with a β-lactamase inhibitor (e.g., piperacillin–tazobactam and ticarcillin–clavulanate), broad-spectrum cephalosporins given with or without a β-lactamase inhibitor (e.g., cefepime, ceftazidime, and ceftolozane–tazobactam), fluoroquinolones (e.g., levofloxacin and ciprofloxacin), carbapenems (e.g., imipenem, doripenem, and meropenem), monobactams (e.g., aztreonam), and aminoglycosides (e.g., tobramycin, gentamicin, and amikacin) [17]. Pseudomonas (PA) often presents with a large array of genetic resistance mechanisms to antibiotics. Multidrug-resistant *P. aeruginosa* (MDR-PA) is defined as *P. aeruginosa* not susceptible to at least one antibiotic in at least three antibiotic classes for which *P. aeruginosa* susceptibility is generally expected. Extensive drug resistance (XDR-PA) is nonsusceptibility to at least one agent in all but one or two antibiotic classes, and pan-drug resistance (PDR-PA) is nonsusceptibility to all agents in all classes [18]. Such drug-resistant microbial strains may complicate the initial medical management of thoracic infections, diverting a percentage of patients towards surgery. Surgery has an established source-control role for these complicated pleural and parenchymal infections in addition to clear surgical indications, namely hemoptysis, pyoptysis due to ruptured abscess, and pulmonary gangrene [4,8,11]. Resection of necrotic lung tissue is frequently necessary, with the extent of surgery determined by the degree of parenchymal destruction and the viability of the remaining lung [19]. Surgery is hampered by various patient-related factors, including poor general condition, respiratory failure, and the extent of lung necrosis, all of which determine the decision and timing for surgery. Both single-stage and multi-stage surgical procedures are considered appropriate, including thoracoplastic procedures. The most typical of the thoracoplastic procedures is open thoracostomy (Eloesser and Clagett’s procedures). This is a procedure from the ‘tuberculosis era’, indicated in persistent pleural sepsis regardless of coexisting parenchymal necrosis. This involves the permanent removal of multiple ribs—typically two to six—creating an open communication to facilitate regular lavage and debridement of the septic cavity to achieve complete sterilization [20]. However, it is not considered a first-step source-control procedure mainly due to its inherently deforming nature and resource-consuming long-term follow-up. Other alternative myoplastic procedures of a lesser degree of deformation have failed to gain popularity among thoracic surgeons as they require specialized training and expertise [21]. Whereas the surgical procedure selection is primarily a thoracic surgeon’s duty, the actual clinical decision at which treatment escalates from closed pleural-space management to surgery (or thoracoplasty) for definitive source control remains unclear. For multi- or pan-resistant strains, the treatment rationale is also underexplored. In this case report, we present a stepwise approach wherein a PDR- PA is identified in a previously operated patient with persistent pleural and parenchymal sepsis, highlighting the point of treatment transition.

## 2. Case Presentation

A 72-year-old male of low socio-economic status, long smoking history and symptoms of community-acquired pneumonia was admitted through the Emergency Department for further treatment. He presented febrile (39.0 °C), tachypnoic (respiratory rate 25/min), with low pulse oximetry (90%) in a 45% O2 Venturi open face mask, and with a baseline chest X-ray revealing right-sided parenchymal infiltrates and a moderate basal pleural effusion compatible with parapneumonic effusion (Figure 1A). Infection was confirmed by increased inflammatory markers (white cell count, 19.32 K/μL; neutrophils, 17.3 K/μL; C-reactive protein [CRP], 417 mg/L) (Figure 2 and Figure 3). On admission, he underwent diagnostic bedside paracentesis, which produced cloudy purulent effusion with pH 7.14 and >145,000 white cells/μL. Due to the purulent nature, an intercostal chest drain (28Fr) was inserted for effective drainage, producing 1100 mL over the first 24 h. Light microscopy of the pleural fluid revealed Gram-positive cocci. We commenced empirical intravenous piperacillin/tazobactam (4.5 g four times daily) while waiting for final microbiology results. Three days later, the first set of pleural fluid cultures was positive for Staphylococcus aureus and Streptococcus canis. (Table 1 and Table 2) The antibiotics were adjusted to intravenous ceftriaxone (2 g) and clindamycin (1.2 g) three times daily. However, clindamycin was discontinued after 2 days due to diarrhea. There was respiratory improvement with pulse oximetry of 97% using a nasal cannula at 6 L/min, but the pyrexia remained unchanged (maximum body temperature 37.9 °C), and there was constant purulent discharge of 200 mL daily from the intercostal chest drain. Inflammatory markers were still raised 5 days post-admission (white cell count, 18.77 K/μL; neutrophils, 16.43 K/μL; CRP, 177 mg/L) (Figure 2 and Figure 3). Surgical drainage was considered to control the pleural sepsis.

Tables of Antimicrobial Sensitivities and MIC.

For Sensitivity Column: S stands for sensitive and R for resistance to the antimicrobial agent.

The numbers in the MIC column indicate the Minimum Inhibitory Concentration value, defined as the lowest concentration of the antibiotic preventing any visible growth of the microorganism obtained from the pleural cavity. (MIC).

The first set of pus cultures was initially obtained from the pleural cavity.

The first surgical intervention 11 days after admission was video-assisted thoracoscopic surgery (VATS) under general anesthesia for pleural cavity debridement, deloculation and partial decortication of the visceral pleura. Postoperative recovery was uneventful regarding bleeding and postoperative pain. The patient’s pyrexia remained unaltered, raising concerns regarding the effectiveness of surgery. Otherwise, his white cell count and neutrophil count decreased to normal (3.4 K/μL and 2.24 K/μL, respectively), whereas CRP remained increased (116 mg/L). In terms of microbiology, postoperative blood cultures were sterile, and interestingly, pleural samples obtained during the surgery were also sterile. Additional urine and fecal samples were negative, and thrombophlebitis was ruled out. Due to ongoing pyrexia with non-diagnostic positive cultures available, the antibiotics were changed on empirical grounds to a broad-spectrum intravenous ampicillin with sulbactam (3 g) four times daily and intravenous metronidazol (500 g) three times daily. On repetitive postoperative chest X-rays (Figure 1B), there was a noticeable increase in pleural fluid collections. Whole-body CT (Figure 4) revealed right basal pleural effusion with NLI of the middle lobe and complete atelectasis of the lower lobe, mandating further surgery for parenchymal and pleural sepsis control.

The second surgical intervention, 25 days post-admission (2 weeks after VATS), was an exploratory right thoracotomy for pleural and parenchymal assessment under general anesthesia. The middle lobe was irreversibly necrotic, so a middle lobectomy was performed without bronchial stump coverage and partial decortication of the visceral pleura with full decortication of the parietal pleura. Further pleural samples were obtained for microbiological and histopathological evaluation (Figure 5). Two wide-bore 36Fr intercostal chest drains were left in situ for pleural drainage. The recovery was uncomplicated, and the last chest drain was removed after 12 days. The cultures of intraoperative pleural samples produced a multi-resistant Gram-negative strain, so the antibiotics were adjusted to a salvage combination of intravenous sodium colistimethate (4,500,000 IU) twice daily in combination with ceftazidime/avibactam (2.5 g) and aztreonam (2 g) three times daily until the exact sensitivities were available.

The exact strain was identified as *Pseudomonas aeruginosa*, which was resistant to all available antibiotics (pan-resistant or PR-PA), as shown in Table 3. The current antibiotic regimen remained a salvage solution due to the unavailability of other options. Colistin was interrupted 3 days after commencement due to nephropathy compatible with acute tubular necrosis. The fever remained steadily present at <38 °C, with raised CRP up to 266 mg/L, but other inflammatory markers remained within the normal range (white cell count, 8.31 K/μL; neutrophils, 6.8 K/μL). Repeat cultures from blood, urine, and fecal samples were sterile. Repetitive chest X-rays (Figure 1D) identified recurrent complex pleural effusion considered to be infected by PR-PA.

A third surgical intervention 60 days post-admission was necessary to address the non-resolving septic pleural space after two decortications and one lobectomy. Under general anesthesia and through a redo-thoracotomy, an open thoracostomy (Eloesser type) was performed by removing the lateral arch of three successive ribs (5th, 6th, and 7th). On the first postoperative day, he became apyrexic. The pleural cavity was irrigated twice daily with povidone solution diluted 50% with normal saline. The antibiotics were stopped immediately from postoperative day 1. A vacuum device was inserted 5 days postoperatively with weekly dressing changes for 8 weeks. The patient recovered completely within 8 weeks. He remained apyrexic throughout his recovery with normal inflammatory markers; CRP decreased gradually to reach a minimum value of 34.7 mg/L. The open thoracostomy wound eventually shrank completely, and by 5 months post-fenestration, there was no open wound (Figure 1F).

## 3. Discussion

This described case of a refractory thoracic infection has no novelties regarding the surgical management. It is accepted by clinicians that complex pleural empyema and NLIs, together or individually presented, require multi-step treatment approaches to prevent fatal sepsis. Although prolonged antimicrobial treatment is the usual approach, repetitive surgical procedures are necessary for resistant and relapse cases [8,10]. The emergence of resistant microbes, especially in their pan-resistant forms, complicates the medical management of thoracic infections on regular respiratory wards. Current advances in respiratory and intensive care medicine often permit adequate sepsis control, rendering major surgery necessary in only select cases [22]. Larose recently described how early assessment of the extent of parenchyma destruction (>50% of the lobe) without distinction between necrotizing pneumonia or gangrene and mechanical ventilation with vasopressor support should dictate treatment in an advanced specialized medical unit. In a cohort of 50 patients, only four (8%) required surgery for pleural decortication, and none required parenchymal resection, with good outcomes [22]. Other authors have reported that early intervention and prior stabilization are associated with favorable outcomes [4,23]. However, in everyday clinical practice, many patients present as already septic and possibly mismanaged from the beginning or already on inappropriate antimicrobial treatments. For them, medical treatment has failed before it is even started, and surgery is often unavoidable. Those already in respiratory distress and on mechanical ventilation carry a higher mortality risk [24].

Definite surgical indications independent of any ongoing medical treatment are hemoptysis and a massive air leak due to bronchopleural fistula due to ruptured lung abscess or extensive necrotic pneumonia [10,25,26]. As these catastrophic infection sequelae are rather rare, the commonest surgical indication is failed medical treatment with persistent sepsis [27,28]. Often, a single procedure is insufficient to provide source control and may even deteriorate sepsis in up to 20% of patients, who eventually require lung resection for NLI [29,30,31]. The stepwise surgical pathway in this case, aimed at effective sepsis control, presented as constant pyrexia lasting for nearly two months. Initially, the least invasive procedure (VATS) for drainage, deloculation and partial decortication was used. This did improve the inflammatory values but not the pyrexia, and underlying pneumonia was thought to still be in progress, eventually developing into necrosis of the middle lobe. At this point, there is no evidence or suspicion of PDR-PA. The second procedure was a thoracotomy, the standard approach for major surgery involving lung resection and associated with higher risks and complications than VATS. In centers specializing in surgery for thoracic infections, any lung resection can be performed safely using VATS, even after previous thoracic procedures [29,30,31,32], but in low-volume centers, it is better to perform standard procedures.

The identification of PDR-PA in the surgical specimen of the second surgical procedure precluded a non-favorable postoperative course. Pleural sepsis remained at peak with evidence that additional surgery was required for debridement. Fortunately, pneumonectomy was avoided, as right-sided pneumonectomies are associated with a higher risk of postoperative bronchial insufficiency and morbidity [33]. Recognizing the enduring, resistant nature of PDR-PA, the decision was made to convert the closed pleural cavity, where there may be limited antibiotic penetration through inflamed tissues, into an open cavity to allow direct access for daily antiseptic management with antiseptic solutions and a vacuum system [34]. Such a plan has to be well explained to the patient and family, as thoracoplasties are deforming procedures with a challenging daily routine, especially during the first days, in which several dressing changes are required. An open thoracostomy, also known as an Eloesser flap, can be performed under conventional two-lung ventilation, though we suggest single-lung ventilation to prevent contralateral lung bronchial spillage [21]. The size of the thoracostomy has to be large enough to allow access to the least accessible pleural sites and prevent new septic loculations. In addition, narrow thoracostomies tend to shrink prematurely before achieving pathogen sterility of the pleural cavity. Reconsidering the sequence of surgical events, this third procedure could have been offered during the middle lobectomy–decortication if PDR-PA was diagnosed prior to the procedure. It is unclear at which point PDR-PA was contacted and if this was in the community setting or during admission. Was it a polymicrobial infection from the initial admission or a superinfection during hospitalization? It was definitely not due to culture-site variation, as both pleural samples were obtained directly from the pleural field. PDR-PA probably represents a nosocomial superinfection contacted at any time point, probably during the initial clinically severe presentation of pneumonia. As there were active surgical problems, we believe that no delay should occur down the treatment pathway. Delaying surgery is a well-recognized negative prognostic parameter in various surgical pathologies, and NLIs are no exception to this rule. Postoperative antibiotic cessation was an individualized approach rather than the common practice. In selected refractory cases with PDR-persistent sepsis, an open thoracostomy may be the only salvage strategy. Whether it should be adopted earlier requires further evidence, and this decision cannot be justified by a single case report. This is the main limitation of our study, despite the fact that in current times, thoracoplastic procedures are still current but largely not preferred by the younger generation of thoracic surgeons. It is unknown if there will be any prospective studies relating surgery to PDR thoracic infections.

## 4. Conclusions

This single case report of a complex thoracic infection treated with multiple procedures of gradual complexity over long-standing sepsis signifies the importance of considering surgery early in the treatment plan of PDR microbial infections, always on a ‘selected-only-cases’ basis. Although intensive and focused medical management in experienced centers may exclude surgery for most cases, by definition, a group of patients will often require more than one surgery of various severities. The selection of the procedure should not be oriented towards minimal invasiveness, as in lung cancer cases, but towards a definite source-control result. As soon as a PDR organism is identified in connection with local or systemic septic manifestation, a multidisciplinary setting should consider aggressive surgery, such as thoracoplasty, as an option in the event of relapse and provide a thorough explanation of the aims and risks to the patient and all involved specialties to avoid delays in the prolonged management of sepsis.

## Figures and Tables

**Figure 1:**
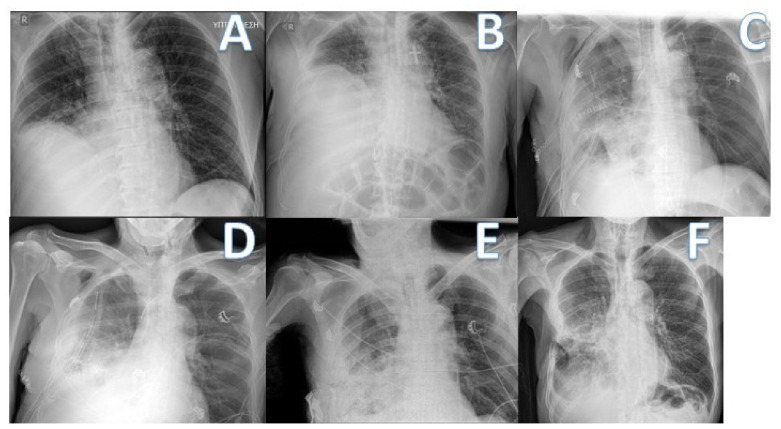
Chest X-ray series from admission through treatment. (**A**) Imaging at admission showed non-specific infiltrates. The moderate pleural effusion was associated with lung atelectasis. (**B**) Post-VATS re-collection of pleural effusion indicating failure of the initial interventions. (**C**) Post-thoracotomy CXR shows adequate lung expansion but moderate basal effusion space or potential empyema recurrence. (**D**) No improvement in pleural empyema despite antibiotics and a chest drain in situ. (**E**) Post-thoracostomy with a betadine-soaked swab in the open pleural cavity. (**F**) Three-month follow-up with adequate lung expansion, absent pleural space, and minimal thoracic deformity.

**Figure 2:**
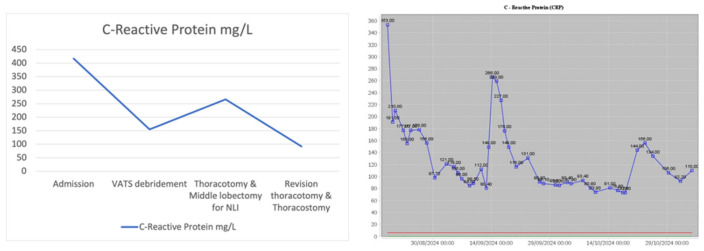
C-Reactive protein trend (in mg/L) over 10 weeks and in relation to the surgical interventions.

**Figure 3:**
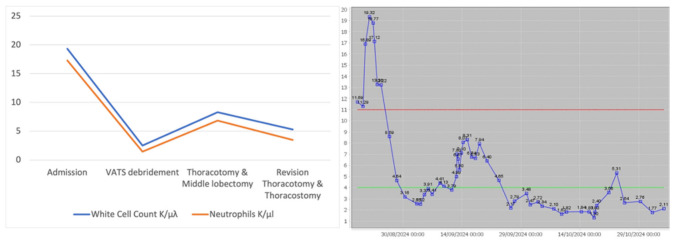
White-cell count trend (in K/μL) over 10 weeks in relation to the surgical interventions.

**Figure 4:**
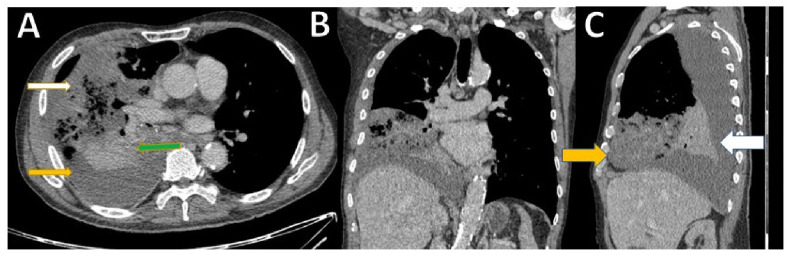
Postoperative computed tomography of the chest. (**A**) The initial intervention failed to control pleural sepsis with additional parenchymal complications, i.e., middle lobe necrosis (white arrow). The green arrow indicates the completely collapsed lower lobe, which intraoperatively proved to be viable. The orange arrow indicates the parapneumonic effusion. (**B**) Coronal view with multiple sub-centimeter parenchymal abscesses of the middle lobe. (**C**) Sagittal view showing middle lobe necrosis (orange arrow) and reversible atelectasis of the lower lobe. The pleural empyema (white arrow) occupies a large intrapleural volume.

**Figure 5:**
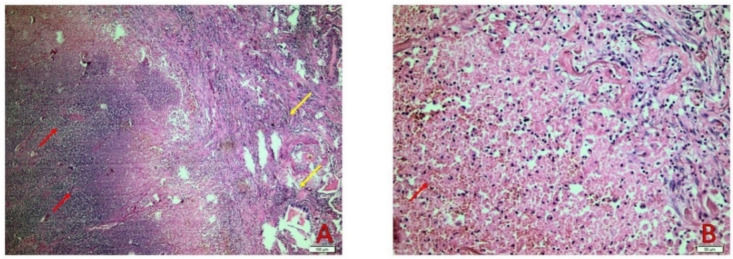
Histopathology of the resected necrotic middle lobe. (**A**) Low-power examination (×40) showing extensive areas of coagulative necrosis (red arrows) next to areas of viable lung parenchyma (yellow arrows). Scale bar: 100 µm. (**B**) Higher power examination (×200) distinguished necrotic ghost cells and hemosiderin deposits (red arrow). Hematoxylin and eosin stain. Scale bar: 50 µm.

**Table 1:** Cultivated organism is Staphylococcus aureus.

Antimicrobial Agent	Sensitivity	MIC
Ceftaroline	S	0.25
Ciprofloxacin	S	≤0.5
Clindamycin	S	≤0.25
Daptomycin	S	0.5
Erythromycin	S	≤0.25
Fosfomycin	S	≤16
Fusidic Acid	S	≤0.5
Gentamicin	S	≤1
Levofloxacin	S	≤0.5
Linezolid	S	2
Moxifloxacin	S	≤0.25
Mupirocin	S	≤0.5
Mupirocin-HL	S	≤256
Oxacillin	S	≤0.25
Penicillin	S	0.125
Pristinamycin	R	-
Quinupristin/Dalfopristin	R	-
Rifampin	R	≤0.25
Teicoplanin	S	≤0.5
Tetracycline	S	≤0.5
Tigecycline	S	≤0.125
Tobramycin	S	≤1
Trimeth/Sulfamethoxazole	S	≤0.5/9.5
Vancomycin	S	1

No testing for: Chloramphenicol, Nitrofurantoin, Trimethoprim.

**Table 2:** Cultivated organism is Streptococcus canis.

Antimicrobial Agent	Sensitivity	MIC
Penicillin	S	≤0.031
Moxifloxacin	S	≤0.25
Levofloxacin	S	1
Clindamycin	R	>0.5
Gentamicin	S	≤250
Tetracycline	R	>4
Vancomycin	S	≤0.5
Teicoplanin	S	≤1
Erythromycin	R	>0.5
Daptomycin	S	≤0.25
Linezolid	S	1

No testing for: Amoxicillin, Cefepime, Cefotaxime, Cefuroxime, Meropenem, Pristinamycin, Trimethoprim/Sulfamethoxazole, Chloramphenicol. Second set of pus cultures obtained two months later from the pleural cavity.

**Table 3:** Cultivated organism is Pseudomonas aeruginosa.

Antimicrobial Agent	Sensitivity	MIC
Aztreonam	R	>16
Piperacillin/Tazobactam	R	>64/4
Cefepime	R	>8
Ceftazidime	R	>16
Ceftazidime/Avibactam	R	>8/4
Ceftolozane/Tazobactam	R	>4/4
Imipenem	R	>8
Meropenem	R	>16
Meropenem/Vaborbactam	R	>8/8
Ciprofloxacin	R	>1
Amikacin	R	>16
Tobramycin	R	>4
Colistin	R	2

No testing for: Amoxicillin/Clavulanate, Ampicillin, Temocillin, Cefoxitin, Ceftriaxone, Cefuroxime, Ertapenem, Fosfomycin, Levofloxacin, Gentamicin, Tigecycline, Colistin, Trimeth/Sulfamethoxazole.

## Data Availability

The data are available upon request due to ethical reasons.
